# Carpe DM: The First Global Diabetes Targets

**DOI:** 10.9745/GHSP-D-22-00403

**Published:** 2023-04-28

**Authors:** Jeremy I. Schwartz, Kaushik Ramaiya, Margo Warren, Prashant Yadav, Grace Castillo, Roshini George, Helen McGuire

**Affiliations:** a Section of General Internal Medicine, Yale University School of Medicine, New Haven, CT, USA.; b Uganda Initiative for Integrated Management of Non-Communicable Diseases, Kampala, Uganda.; c Coalition for Access to NCD Medicines and Products.; d Department of Internal Medicine, Shree Hindu Mandal Hospital, Dar es Salaam, Tanzania.; e Access to Medicine Foundation, Amsterdam, The Netherlands.; f Center for Global Development, Washington, DC, USA.; g Technology and Operations Management, INSEAD, Fontainebleau, France.; h PATH, Seattle, WA, USA.

## Abstract

The authors discuss the newly adopted global diabetes targets and their potential role in driving funding, advocacy, research, and clinical care to reduce the massive global disparities in access to quality diabetes care.

## Key Messages

- Diabetes control is hampered by global inequities in care, which the new global diabetes targets aim to address.
- There are notable similarities and differences between the diabetes targets and the widely adopted HIV targets.
- These targets, combined with concrete operational plans, will help the global community focus efforts toward achieving global diabetes control.

## GLOBAL COVERAGE TARGETS FOR DIABETES

In May 2022, the 75^th^ World Health Assembly adopted 5 groundbreaking global targets for diabetes mellitus (DM) for 2030.^1^

1. 80% of people living with DM are diagnosed
2. 80% of those diagnosed achieve glycemic control
3. 80% of those diagnosed achieve blood pressure control
4. 60% of people with DM aged 40 years or older receive statins
5. 100% of people with type 1 DM have access to affordable insulin and blood glucose self-monitoring.

The Global Diabetes Compact, which launched in 2021 on the centennial of insulin’s discovery, encompasses these targets. The Compact’s vision is to reduce the risk of diabetes and ensure everyone diagnosed with diabetes has access to equitable, comprehensive, affordable, and quality treatment and care.^2^

The global prevalence of type 2 DM is expected to increase from 537 million in 2021 to 643 million in 2030 and to 783 million by 2045. The prevalence in sub-Saharan Africa will increase by 134% in that period, far more dramatically than in any other region.^3^ Vast global inequities shape access to quality DM prevention and care.^4^ Pharmaceutical access programs for DM medicines vary greatly in their reach, from less than 100 persons living with DM in some low-income countries to more than 1.6 million in some lower-middle-income countries.^5^ In the United States, 1 in 4 people with insulin-dependent DM ration insulin due to cost,^6^ a rate comparable to most low- and middle-income countries, while in other high-income countries, like the United Kingdom, cost-related insulin underuse is virtually nonexistent.^7^ Improving access to insulin over the next decade is expected to save more than 330,000 disability-adjusted life-years.^8^ The largest of these gains would be seen in sub-Saharan Africa, where most persons living with DM first come to medical attention with complications from underdiagnosis and poor glycemic control.^9^ Resource variability also deepens these challenges and lessens people’s ability to consistently engage in DM self-care.^10^ These inequities present an opportunity for global attention and focus.

Targets present quantifiable milestones to the global community and provide a focus for coalescing the efforts of multiple stakeholders. As we have seen with the widely adopted Joint United Nations Programme on HIV/AIDS “90-90-90” HIV targets,^11^ these targets can become ubiquitous goalposts for global, regional, and national monitoring of program quality. Targets inform policymaking and form the pillars of care cascades, such as those for HIV or hypertension.^12^ While the HIV targets are directed at HIV-specific indicators—diagnosis, treatment, and viral suppression—the DM targets are, appropriately, cross-cutting. Attention to blood pressure and lipid management recognizes the common risk factors underlying DM and cardiovascular disease (CVD). CVD, the leading cause of death globally,^13^ can occur 10–15 years earlier in persons living with DM compared to those without DM.^14^ While HIV has been addressed through a vertical, or disease-specific approach, driven by targeted global funding mechanisms and bolstered by a passionate global advocacy movement, the control of DM and other noncommunicable diseases must be addressed through a comprehensive strategy of integrated approaches and care delivery models. However, without similar multilateral funding to contribute to market-shaping and developing systems for the procurement of tools to diagnose, monitor, and treat DM, achieving these targets is more challenging.

The control of DM and other noncommunicable diseases must be addressed through a comprehensive strategy of integrated approaches and care delivery models.

## ALIGNING TARGETS WITH OPERATIONAL PLANS TO ACHIEVE GOALS

Clear consensus-based targets, combined with aligned operational plans across infectious and noncommunicable diseases, are crucial to making progress in this arena. DM prevention, treatment, and risk reduction require coordinated resources, decisions, and actions from an array of stakeholders—donors, the private sector (including pharmaceutical companies and other health product developers and manufacturers), government, health care providers, and people living with DM. However, this vital coordination can only occur when collectively accepted targets shape concrete plans for financing, procurement, distribution, and dispensing of cost-effective DM and CVD products. One strategy—regional pooled procurement—could accelerate access to key health products. However, governments will need to look for sustainable procurement solutions, and the private sector will need to collaborate to improve access to their products. Many countries do not provide coverage for noncommunicable diseases, even within universal health coverage schemes. This poses a key challenge for access to medicines and medical devices, resulting in great financial burden for persons living with DM. It would be deceptive to anticipate that resource-constrained countries will be able to fully cover such costs. Extending the impact of universal health coverage programs requires a multisectoral, person-centered approach that includes strengthening primary health care and community health worker programs; bolstering health care worker training; simple treatment protocols; and task sharing that moves the multiple facets of prevention and management away from doctors and facilities towards other clinical staff, people living with DM by way of self-care, and the community.^15^

While these targets do address key clinical metrics for quality DM care and CVD risk reduction, they lack any focus on DM prevention. The World Health Organization’s “best buys” are evidence-based, cost-effective interventions that cost under $100 per disability-adjusted life-year averted in low- and middle-income countries.^16^ Drug therapy for those with DM and/or hypertension and a high risk of CVD is a best buy. But so too are upstream preventive efforts, including reducing physical inactivity and unhealthy diet, that are widely known to prevent or delay the onset of DM^17^ or even lead to its remission.^18^ Such prevention measures will require multisectoral approaches that could include mass media educational campaigns, reformulation of food products, and urban planning to encourage physical activity through changes to the built environment.^17^

## CONCLUSION

We call on the global community—persons living with DM and their advocates, policymakers, pharmaceutical industry, public health and health care practitioners, and researchers—to “seize the day.” As we move forward to the September 2023 United Nations High-Level Meeting on Universal Health Coverage, let’s use the DM targets as an opportunity to drive a new generation of advocacy, policy, prevention, care, and implementation research to inform affordable, practical, locally relevant, and contextualized health service delivery solutions to reduce the risk of DM and its complications worldwide…Carpe DM!

## Notes

**Peer Reviewed**

**First published online:** April 3, 2023.
